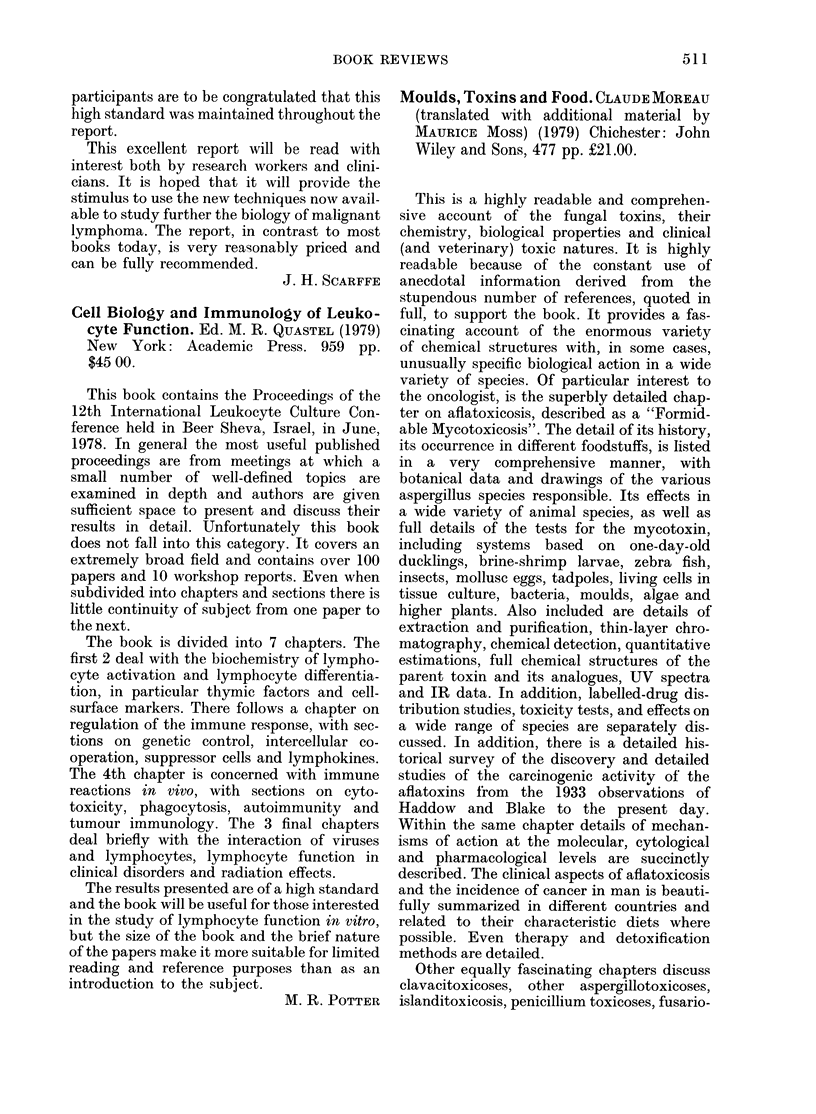# Cell Biology and Immunology of Leukocyte Function

**Published:** 1980-03

**Authors:** M. R. Potter


**Cell Biology and Immunology of Leukocyte Function.** Ed. M. R. Quastel (1979) New York: Academic Press. 959 pp. $45 00.

This book contains the Proceedings of the 12th International Leukocyte Culture Conference held in Beer Sheva, Israel, in June, 1978. In general the most useful published proceedings are from meetings at which a small number of well-defined topics are examined in depth and authors are given sufficient space to present and discuss their results in detail. Unfortunately this book does not fall into this category. It covers an extremely broad field and contains over 100 papers and 10 workshop reports. Even when subdivided into chapters and sections there is little continuity of subject from one paper to the next.

The book is divided into 7 chapters. The first 2 deal with the biochemistry of lymphocyte activation and lymphocyte differentiation, in particular thymic factors and cell-surface markers. There follows a chapter on regulation of the immune response, with sections on genetic control, intercellular co-operation, suppressor cells and lymphokines. The 4th chapter is concerned with immune reactions *in vivo*, with sections on cytotoxicity, phagocytosis, autoimmunity and tumour immunology. The 3 final chapters deal briefly with the interaction of viruses and lymphocytes, lymphocyte function in clinical disorders and radiation effects.

The results presented are of a high standard and the book will be useful for those interested in the study of lymphocyte function *in vitro*, but the size of the book and the brief nature of the papers make it more suitable for limited reading and reference purposes than as an introduction to the subject.

M. R. Potter